# RP-CAD for Lipid Quantification: Systematic Method Development and Intensified LNP Process Characterization

**DOI:** 10.3390/ph17091217

**Published:** 2024-09-16

**Authors:** Nicole Beckert, Annabelle Dietrich, Jürgen Hubbuch

**Affiliations:** Institute of Process Engineering in Life Sciences—Section IV: Biomolecular Separation Engineering, Karlsruhe Institute of Technology (KIT), 76131 Karlsruhe, Germany; nicole.beckert@kit.edu (N.B.); annabelle.dietrich@kit.edu (A.D.)

**Keywords:** lipid nanoparticles, charged aerosol detection, power function value, reversed-phase chromatography, bioprocessing, method validation, intensification

## Abstract

Lipid nanoparticles (LNPs) and their versatile nucleic acid payloads bear great potential as delivery systems. Despite their complex lipid composition, their quality is primarily judged by particle characteristics and nucleic acid encapsulation. In this study, we present a holistic reversed-phase (RP)-charged aerosol detection (CAD)-based method developed for commonly used LNP formulations, allowing for intensified LNP and process characterization. We used an experimental approach for power function value (PFV) optimization termed exploratory calibration, providing a single PFV (1.3) in an appropriate linearity range for all six lipids. Followed by the procedure of method calibration and validation, linearity (10–400 ng, *R*^2^ > 0.996), precision, accuracy, and robustness were effectively proven. To complement the commonly determined LNP attributes and to evaluate the process performance across LNP processing, the developed RP-CAD method was applied in a process parameter study varying the total flow rate (TFR) during microfluidic mixing. The RP-CAD method revealed a constant lipid molar ratio across processing but identified deviations in the theoretical lipid content and general lipid loss, which were both, however, entirely TFR-independent. The deviations in lipid content could be successfully traced back to the lipid stock solution preparation. In contrast, the observed lipid loss was attributable to the small-scale dialysis following microfluidic mixing. Overall, this study establishes a foundation for employing RP-CAD for lipid quantification throughout LNP processing, and it highlights the potential to extend its applicability to other LNPs, process parameter studies, or processes such as cross-flow filtration.

## 1. Introduction

As the potential of small interfering RNA (siRNA) for gene silencing was revealed a few decades ago [1], lipid-based delivery systems—flexible in their structural complexity and composition—evolved in parallel, unlocking the full potential of lipid-based siRNA delivery, which was recently demonstrated by the approval of Onpattro (patrisan) [2].

For the encapsulation of nucleic acid payloads, as well as for the nucleic acid release, the role of a cationic lipid (CL) is crucial as anionic siRNA has been shown to be complexed with the CL [3]. The CL is part of a delivery system called lipid nanoparticle (LNP) with three additional lipids, namely cholesterol, a phospholipid, and a polyethylene glycol lipid (PEGL). The structure of LNPs has already been visualized by transmission electron microscopy (TEM) and exhibits non-continuous lipid bilayers with an electron-dense core [3,4].

As each lipid component in LNPs serves distinct purposes [5], lipid selection, as well as their lipid molar ratio, are crucial for proper functionality. Phospholipid content, cholesterol content, and phospholipid type affect LNP morphology [6], while variations in size have been observed for different CLs [7]. Since the concentration of PEGL constitutes the smallest proportion within an LNP, a precise adjustment of its content is critical due to its influence on LNP size, as well as on the prevention of inter-particle fusion [8,9,10], the circulation time, and activity in vivo [11]. The role of lipid components in LNPs was comprehensively summarized by Albertsen et al. [5]. To fulfill these varieties of functions, the incorporated particular lipid content in LNPs, termed the lipid molar ratio, has been widely reported within a similar range. For siRNA-LNPs, with a lipid molar ratio of 50:10:38.5:1.5 mol% (CL:phospholipid:cholesterol:PEGL), potency was early on verified in vivo [12,13,14], and, consequently, this ratio has been extensively utilized in fundamental research and development [8,15,16,17,18,19,20]. Thus far, this ratio has not only demonstrated efficacy in Onpattro (patrisan), being the first LNP approved by the FDA, but it was also employed for the messenger RNA (mRNA)-1273 vaccine during the COVID-19 pandemic [2].

Besides the impact of lipids on a molecular basis, the impact of the production and purification parameters on the physicochemical properties of LNPs has been investigated in the literature, hereafter referred to as parameter studies. Microfluidic mixing devices enable the reproducible and scalable mixing of the lipid-containing organic and the nucleic acid-containing aqueous phase, inducing LNP formation [4]. Key microfluidic process parameters, such as total flow rate (TFR) and flow rate ratio (FRR) [7,21,22], along with aqueous phase characteristics [7,21] and lipid concentration in the organic phase [21,22], have been shown to significantly influence particle size and polydispersity. The N/P ratio describing the molar ratio of positively charged nitrogen (N) within the amines of the charged lipid to the negatively charged phosphate (P) groups in the nucleic acid backbone has been found to affect LNP morphology [17] and the encapsulation efficiency of nucleic acids [10,21]. Since nucleic acids are commonly dissolved in buffers with a pH of 4–5, microfluidic mixing is typically followed by dialysis to neutralize pH and remove the remaining organic solvent. Except for the pH variations of formulation buffers studied by Terada et al. [22], the impact of dialysis as a purification step remains rather unexplored.

To summarize, LNP quality attributes imply characteristics regarding the intact LNP, as well as the nucleic acids and lipids themselves. Although a variety of analytical techniques quantitatively assessing these characteristics exist, as comprehensively reviewed by Fan et al. [23], only a restricted set of analytical methods has been consistently utilized in the context of parameter studies [7,10,21,22]. In particular, LNP physicochemical property assessment comprises surface charge using electrophoretic light scattering (ELS), particle size, and the polydispersity commonly measured by dynamic light scattering (DLS). Additionally, the encapsulation efficiency of nucleic acids in LNPs is determined using fluorescence-based assays. However, lipid content analysis to quantify all lipid components and determine the lipid molar ratio is not routinely conducted, especially not across LNP processing.

High performance liquid chromatography (HPLC) approaches are deployed for lipid separation and quantification across their molecular diversity. Reversed-phase (RP)-HPLC serves for the separation of lipid molecular species according to their relative hydrophobicity that arises from differences in head group polarity, tail unsaturation, and alkyl chain length [24]. As most lipids lack chromophores, typical spectrophotometric detection for lipid quantification is not applicable. Here, charged aerosol detection (CAD) can be deployed, where the mobile phase is nebulized and dried, the formed particles are positively charged by a stream of nitrogen, and the charge is measured by a highly sensitive electrometer [25,26]. CAD outperforms the other widespread aerosol-based evaporative light scattering detection in terms of sensitivity and width of the dynamic range [27]. Both performance parameters can be further improved by optimizing the built-in power function value (PFV), which linearizes the detector’s response. Here, several strategies to determine the optimal PFV were explored, including experimental, empirical, and mathematical approaches [28,29,30,31]. These studies exclusively optimized the PFV for one particular substance, while the literature lacks PFV optimization studies considering multi-component systems such as LNPs.

Thus far, the developed RP-CAD methods for various components of LNPs comprise the lipid quantification of phospholipids [29], PEGLs [32,33], liposomes [34], LNPs [19,20,35,36,37], and further stability studies to monitor the degradation products of the prior listed components [29,33,34,36]. Considering the RP-CAD method application on LNP formulations, different objectives were pursued besides method validation. Li et al. [35] focused on the variation of key chromatographic parameters critical for separation such as the stationary phase, ion-pair agent, and column temperature, while Kinsey et al. [36] and Bender et al. [20] further studied forced lipid degradation. For method development, Kim et al. [19] pursued an alternative strategy, employing an analytical quality-by-design approach. However, these studies proved their methods with solely final formulated LNPs and, except for Kim et al. [19] and Bender et al. [20], with partly unknown lipids and lipid molar ratio specifications. Thus far, no analytical method has been employed across processing nor has the effect of process parameter variations on lipid-related LNP attributes been considered.

In this study, we present a novel holistic RP-CAD method for lipid quantification across LNP production and processing. Based on an experimental approach, we systematically varied the PFV using a simplified lipid stock solution set, focusing on identifying a single PFV for multiple lipids. By expanding the stock solution set for method calibration and validation studies, we demonstrated adequate linearity, precision, accuracy, and robustness according to the International Council for Harmonisation (ICH) Q2(R2) guideline [38], enabling lipid quantification in pure and mixed lipid solutions. To demonstrate the RP-CAD method’s applicability for quantifying lipids at various stages of the LNP process regardless of specific process parameters, the impact of the TFR as a production parameter on the LNP attributes was studied using LNPs encapsulating a DNA-based model-siRNA. In addition to the commonly determined attributes, particle size, surface charge, and nucleic acid encapsulation, we simultaneously assessed lipid quantities and lipid molar ratio with the newly developed RP-CAD method. These lipid quantities allow for the calculation of lipid recoveries, providing an indicator of the process performance in LNP purification and thus enabling intensified process characterization.

## 2. Results

### 2.1. Lipid Quantification—Method Development, Calibration, and Validation

The RP-CAD method was developed for cholesterol; the phospholipid 1,2-Dioctadecanoyl-sn-glycero-3-phosphocholin (DSPC); two CLs: 1,2-Dioleoyl-3-trimethylammonium-propane (chloride salt) (DOTAP) and N-(4-carboxybenzyl)-N,N-dimethyl-2,3-bis(oleoyloxy)-propan-1-aminium (DOBAQ); two PEGLs: 1,2-Dimyristoyl-rac-glycero-3-methoxypolyethylene glycol-2000 (DMG-PEG) and 1,2-Dimyristoyl-sn-glycero-3-phosphoethanolamine-N-[methoxy(polyethylene glycol)-2000] (ammonium salt) (DMPE-PEG).

#### 2.1.1. Method Development

**Figure 1:**
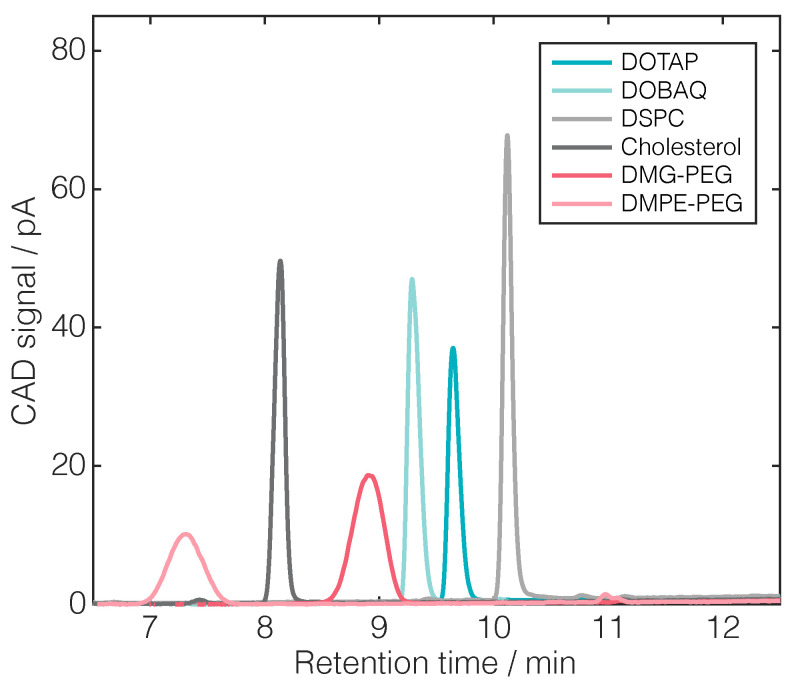
Overlaid HPLC chromatograms of the common lipids used in the LNP formulations: DOTAP, DOBAQ, DSPC, cholesterol, DMG-PEG, and DMPE-PEG.

For lipid separation, RP-CAD solvents and method settings, including column temperature, CAD evaporation temperature, injection volume, flow rate, and gradient design, were determined in preliminary experiments. Figure 1 shows the RP-CAD chromatograms of all the used lipids with a lipid quantity of 400 ng on the column at a PFV of 1.3.

Overall, baseline separation was achieved for all lipids. Considering peak shapes, wider and less sharp peaks were observed for PEGLs compared to other lipid types, which can be attributed to the distribution of the molecular weights within the polyethylene glycol (PEG) chains. Moreover, the differences in the peak areas for equivalent lipid quantities on the column emphasize the importance of employing lipid-specific calibration.

**Figure 2:**
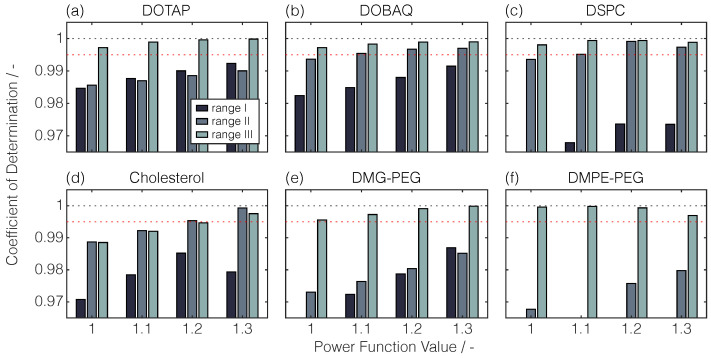
Illustration of the variations in linearity range and PFV. Coefficients of determination ( $R^{2}$ ) are shown for all exploratory calibration curves for the lipids DOTAP (**a**), DOBAQ (**b**), DSPC (**c**), cholesterol (**d**), DMG-PEG (**e**), and DMPE-PEG (**f**). The PFVs were varied between 1.0–1.3 in 0.1 increments. The linearity ranges I, II, and III differed in their maximum lipid quantity on column: 2400, 1200, and 600 ng. $R^{2}$ values above 0.995 represent adequate linearity, marked here with red dashed horizontal lines. For visual purposes, the $R^{2}$ values below 0.965 are not depicted.

**Figure A1:**
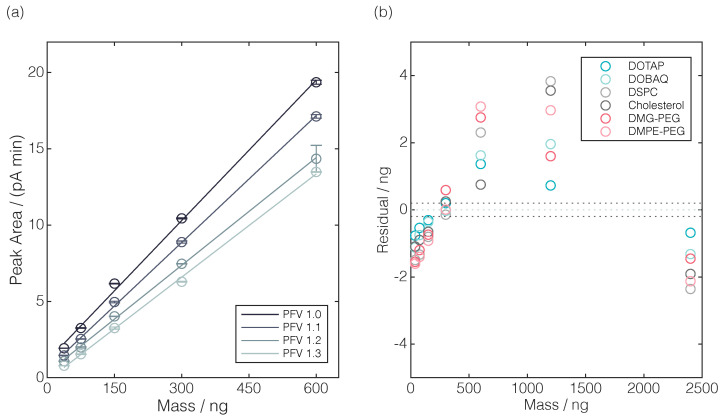
Variation of the PFV and linearity range. The exploratory range I calibration curves (**a**) represent the peak area changes with lipid quantity for the varying PFVs (1.0, 1.1, 1.2, and 1.3) that are exemplarily visualized for DSPC. (**b**) Residual plots for exploratory range III calibration curves and PFV = 1.3 are displayed for the lipids DOTAP, DOBAQ, DSPC, cholesterol, DMG-PEG, and DMPE-PEG. The target residual range of ±0.2 ng is displayed with dashed horizontal lines.

To maintain the linearity between the injected lipid quantity and peak area, preliminary experiments were performed to find an appropriate linearity range in combination with the built-in PFV. To cover wide lipid mass ranges for all lipids while maintaining a manageable level of effort, a simplified lipid stock solution set with a limited number of replicates (*n* = 2, *m* = 2) and data points (*p* ≤ 7) was deployed for exploratory calibration. Despite the three investigated linearity ranges, increased PFVs resulted in decreased peak areas for the same lipid quantity on column (Figure A1a). For the exploratory calibration curves in the three linearity ranges, the $R^{2}$ values are illustrated in Figure 2 for all lipids.

Comparing the linearity ranges, the range with the lowest maximum lipid quantities on column, termed range III, showed the highest $R^{2}$ for almost all tested lipids and PFVs. Except for DOBAQ, DOTAP, and cholesterol, ranges I and II with higher maximum lipid quantities than 600 ng showed insufficient linearity as $R^{2}$ values larger than 0.995 were defined for adequate linearity. In both of these ranges, the residuals were not evenly distributed, as exemplarily shown for PFV = 1.3 (Figure A1b).

By comparing the selected PFVs, higher PFVs resulted in comparable or even higher $R^{2}$ values for DOTAP, DOBAQ, cholesterol, and DMG-PEG, while for DMPE-PEG, a decreased linearity with increasing PFV was observed. Interestingly, when comparing the $R^{2}$ decrease with the peak shape, sharper peaks demonstrated better correlations across broader lipid mass ranges. It has to be noted that a single PFV has to be selected for the RP-CAD method. As $R^{2}$ values larger than 0.995 were obtained at a PFV of 1.3 for all tested lipids, this setting was selected in combination with the linearity range III.

As part of the method development, this strategy of exploratory calibration using a simplified lipid stock solution set with a limited number of replicates (*n* = 2, *m* = 2) and data points (*p* ≤ 7) proved to find a single PFV for all lipids in combination with an appropriate linearity range.

#### 2.1.2. Method Calibration and Validation

To provide a broader basis for method calibration and validation, new measurements were performed within the selected linearity range by expanding the number and type of replicates (*n* = 3 & *m* = 2) and the number of data points (*p* = 9) for the calibration curves.

The RP-CAD method was evaluated regarding the linearity in the range of 10–400 ng lipids on column, precision, accuracy, and robustness. Besides the retention times, the regression parameters slope, y-intercept, $R^{2}$ , and Sy.x are listed in Table 1 for all lipids.

**Figure A2:**
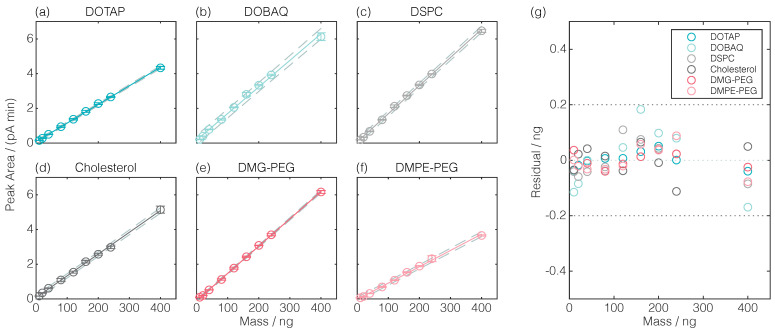
Method calibration—linearity. Changes in the peak area with lipid quantity at PFV = 1.3 are displayed as nine-point calibration curves with their standard deviation for DOTAP (**a**), DOBAQ (**b**), DSPC (**c**), cholesterol (**d**), DMG-PEG (**e**), and DMPE-PEG (**f**). The 95% confidence intervals are displayed as dashed lines. Linear regression equations and coefficients of determination ( $R^{2}$ ) are provided in Table 1. The corresponding residuals (**g**) are within the target residual range of ±0.2 ng and are visualized by dashed horizontal lines.

**Figure A3:**
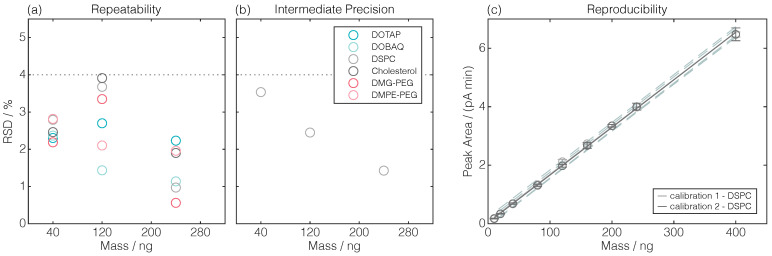
Method validation—precision. The percentage of the residual standard deviation versus lipid quantity is shown for repeatability (**a**) and intermediate precision for DSPC (**b**). The calibration curves for DSPC depicted in (**c**) show the reproducibility of the method.

The mean peak areas with corresponding standard deviations, the linear regression, and the 95% confidence intervals are additionally depicted in Figure A2a–f. The linearity criterion was met with obtained $R^{2}$ values equal to or higher than 0.995, Sy.x below 0.015, and with evenly distributed residuals (Figure A2g) for all lipids. Precision was demonstrated by repeatability, intermediate precision, and reproducibility. Residual standard deviations less than 4% were observed for repeatability (Figure A3a) and intermediate precision (Figure A3b). No statistically significant deviations were obtained for repeated calibrations of DSPC, proving the reproducibility of the method (Figure A3c). Accuracy was displayed by recoveries of 96%, 98%, and 101% for 40, 120, and 240 ng DSPC, respectively.

**Figure 3:**
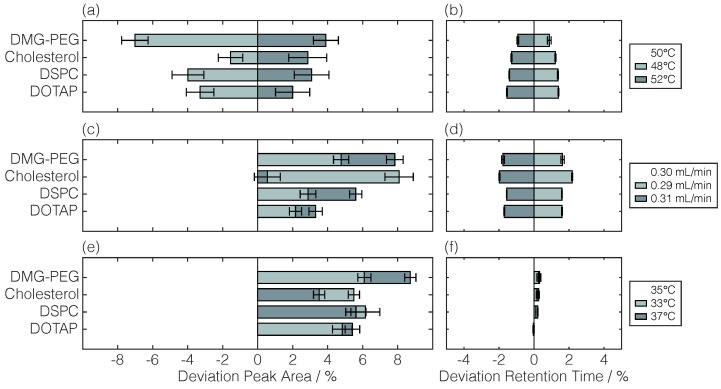
Robustness assessment. The impact of variations in the column oven temperature (**a**,**b**), flow rate (**c**,**d**), and CAD evaporation temperature (**e**,**f**) are shown as a percentage deviation from the standard peak area and retention time per lipid. Standard errors were determined by uncertainty propagation. For visual purposes, the standard method parameters are listed on the top of the legend entries.

Robustness was assessed by minor variations in either the column oven temperature, flow rate, or CAD evaporation temperature using lipid mixtures. Lipid-specific deviations in the peak area and retention time compared to those of standard method parameters are illustrated in Figure 3.

Inverse trends were observed for the peak areas and retention times by column oven temperature variations as smaller peak areas were accompanied by higher retention times for lower temperatures and vice versa (Figure 3a,b). Conversely, and despite parameter in- or decrease, all parameter variations in the flow rate and CAD evaporation temperature resulted in increased peak area deviations from the standard method up to 9% (Figure 3c,e). Regarding retention times, similar trends were observed for both column oven temperature and flow rate variations as the retention time increased by higher parameters and vice versa (Figure 3b,d). Here, deviations up to ±2% were found, while deviations of <0.3% were observed for CAD evaporation temperature variations (Figure 3f). Overall, the observed changes by method parameter variations revealed the sensitivity of the detector’s response, underlining the need for reliable HPLC systems.

In addition, this robustness assessment further confirmed the lipid quantification in lipid mixtures—various lipids were present in 100% organic solvent—compared to pure lipid solutions. Moreover, the analysis of the LNP process solutions still containing aqueous buffer components and nucleic acids could be demonstrated by appropriate sample dilution with ethanol.

In summary, this novel holistic RP-CAD method was thoroughly calibrated and validated for the quantification of specific lipids in lipid mixtures. Further, this method will find its application for lipid quantification across LNP processing.

### 2.2. Applicability for Lipid Nanoparticle Process Characterization

A process parameter study was performed to demonstrate the applicability of the newly developed RP-CAD method for molar ratio and lipid concentration measurements, as well as for lipid recovery. The TFR was varied to evaluate its impact as an isolated production parameter on the LNP attributes across production, processing, and short-term storage. Besides the RP-CAD analytic, measurements for particle size, surface charge, and nucleic acid encapsulation were employed and collectively termed the standard analytical panel.

#### 2.2.1. Standard Analytical Panel

**Figure 4:**
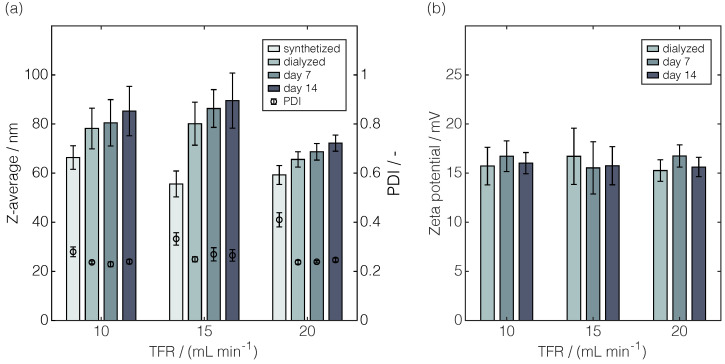
Comparison of the Z-average, PDI, and zeta potential of LNPs, which were produced at different TFRs. The produced LNPs were dialyzed and stored for up to 14 days at 2–8 °C. The Z-average and corresponding PDI (**a**) and zeta potential (**b**) were measured twice from independently diluted samples.

Figure 4 illustrates changes in the Z-average, polydispersity index (PDI), and zeta potential across processing and storage. Together, these three attributes provided a comprehensive impression of the colloidal stability of the particles.

Regardless of the TFR, the Z-average of the particles increased during processing and storage, with a more pronounced increase observed during processing (Figure 4a). The lowest Z-average increase was identified for 20 mL min^−1^ TFR, which was accompanied by the smallest standard deviations, as well as the overall lowest Z-average with 72.2 nm after storage for 14 days. It has to be noted that the highest Z-average values were observed for 15 mL min^−1^ TFR, contradicting the trend expectation from the literature where higher TFRs were anticipated to result in smaller particle sizes [7,21]. However, the trend of smaller particles for higher TFRs could clearly be demonstrated by assessing the significance for 10 and 20 mL min^−1^ TFRs.

**Figure A4:**
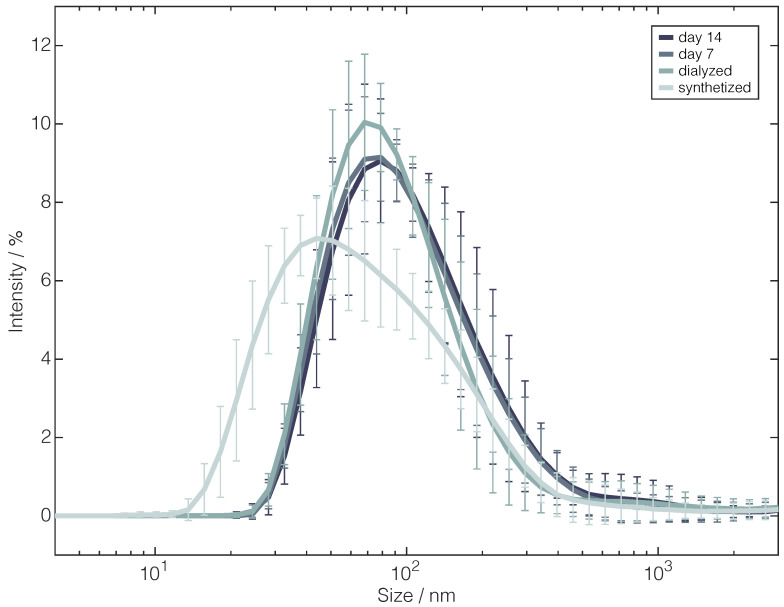
Intensity-weighted size distribution. The percentage intensity versus particle size is shown for the synthesized, dialyzed, and stored LNPs.

An opposite trend was observed for the PDI, showing decreasing values across processing, whereas they remained constant during storage (Figure 4a). Comparing the PDI across the TFRs after synthesis, higher PDIs were identified for higher TFRs. For both 15 and 20 mL min^−1^ TFRs, PDIs above 0.3 were observed with 0.33 and 0.41, respectively. However, after dialysis, lower but comparable PDIs were recorded with values between 0.24–0.25 regardless of the TFR. During storage, all of these PDI measurements exhibited smaller standard deviations compared to those during synthesis, except for the 15 mL min^−1^ TFR where, concurrently, the highest Z-averages were observed. The intensity-weighted size distribution exemplarily shown for the 15 mL min^−1^ TFR (Figure A4) provided a representative illustration of the particle size distribution leading to the Z-average and PDI.

Overall, the observed zeta potentials of approximately 16 mV showed no statistic significance regarding both the TFR and time point of analysis (Figure 4b), indicating stable surface charges during processing and storage. Note that conducting zeta potential measurements was not feasible for synthesized LNPs due to the presence of ethanol.

Besides the colloidal stability in terms of surface charge and particle size, the percentage of encapsulated nucleic acids was determined during processing for each TFR. Encapsulation efficiencies for the synthesized and dialyzed LNPs are listed in Table 2. Across the varied TFRs, encapsulation efficiencies between 97–100% were achieved. No clear differences were observed comparing the encapsulation efficiencies over varied TFRs and across processing. The stability of the free annealed primers, used as model-siRNAs for encapsulation, against Triton™ X-100 was confirmed beforehand through a fluorescence assay that was sensitive for double-stranded DNA (data not shown). This observation points out the absence of model-siRNAs degradation by Triton™ X-100 for encapsulation efficiency determination, ensuring the validity of the encapsulation efficiencies obtained.

In summary, the application of the standard analytical panel (i) proved a stable surface charge of the LNPs and near-complete nucleic acid encapsulation; (ii) revealed the dependencies between the TFRs during LNP synthesis and size, as well as PDI; and (iii) entirely verified the TFR-independent changes of these LNP attributes during production, processing, and short-term storage.

#### 2.2.2. Charged Aerosol Detection for Lipid Quantification and Process Performance

**Figure 5:**
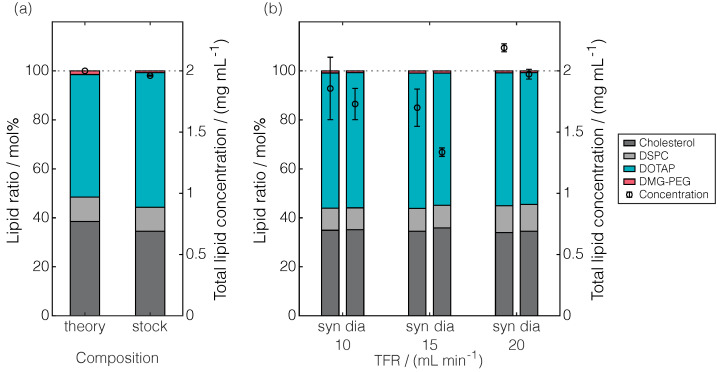
Comparison of the theoretical and measured lipid molar ratio and total lipid concentration. The lipid molar ratio and total lipid concentrations are illustrated as stacked bars and circles, respectively, in the theory and of the stock solution (**a**), and of the LNPs synthesized (syn) at 10, 15, and 20 mL min^−1^ TFR, as well as those dialyzed (dia) (**b**). The theoretical total lipid concentration of 2 mg mL^−1^ is marked as a dashed horizontal line.

To complement the LNP attributes by lipid characteristics, the developed RP-CAD method was applied. Figure 5 illustrates the LNP composition in terms of the lipid molar ratio and the total lipid concentration prior to the process parameter study and during production and processing.

When comparing the theoretical lipid molar ratio with that of the lipid stock solution (Figure 5a), the DOTAP content exceeded the theoretical content of 50 mol%, while lower contents of cholesterol, DSPC, and DMG-PEG than 38.5, 10, and 1.5 mol% were observed, respectively. Considering the relative deviations, DMG-PEG showed the highest deviations from the nominal molar content of approximately 50%.

When comparing the lipid molar ratios during production and processing (Figure 5b), comparable lipid molar ratios were observed regardless of the processing states synthesized or dialyzed and those across the varied TFR. Here, the lipid molar ratio with the 20 mL min^−1^ TFR closely resembled that of the lipid stock solution. Interestingly, despite their different processing states—whether synthesized or dialyzed—the lipid molar ratios at the selected TFRs were even more similar. However, all these lipid molar ratios differed from the theoretical lipid molar ratio in the same manner. In summary, the RP-CAD method revealed deviations from the theoretical lipid molar ratio already existing in the stock solution, which indicates a loss in the totality of lipids and not a loss of certain lipid types over processing.

Considering the total lipid concentration, all of the experiments were designed to reach a target concentration of 2 mg mL^−1^ lipids, which is illustrated by a dashed horizontal line in Figure 5. Diluting the lipid stock solution to an equal extent, a total lipid concentration of 1.96 mg mL^−1^ was obtained (Figure 5a). Note that all lipid concentrations during production and processing (Figure 5b) were calculated based on a constant volume due to appropriate sample dilutions with ethanol during RP-CAD sample preparation. When comparing the total lipid concentrations of synthesized LNPs, they were found to considerably differ by scattering around the theoretical lipid concentration. Here, deviations of approximately 10% accompanied by high standard errors were observed. Correlations between the standard error observations and the TFRs were not apparent. Throughout all the TFRs, a reduction in the total lipid concentrations was visible across the processing states—synthesized to dialyzed—indicating a dilution effect during dialysis or lipid loss. Here, the largest concentration deviation of approximately 22% occurred for the 15 mL min^−1^ TFR, which already represented the condition with the lowest total lipid concentration after synthesis. Conclusively, the RP-CAD method revealed deviations from the theoretical lipid concentration, which are attributable to either the production (synthesis) or processing (dialysis) step regardless of the examined TFR.

To evaluate the process performance of purification by dialysis, lipid recoveries were determined based on the lipid quantities prior to and after dialysis. The densities of the respective ethanol-to-water content were considered for the lipid recovery rates. The lipid recoveries ranged between 88.6 ± 1.2%–100.4 ± 7.6%, with the highest recovery observed for 10 mL min^−1^ TFR. In general, lower lipid recoveries represent lipid loss during dialysis, which implies a loss in LNPs. In summary, the RP-CAD method builds a foundation to determine the process performance of LNP purification, which is exemplarily shown here for purification by dialysis.

## 3. Discussion

The objectives of this work were to develop a RP-CAD method to quantify all lipids in LNPs simultaneously and to apply this method across LNP processing—hence, providing novel, lipid-related LNP attributes and process insights.

### 3.1. RP-CAD for Lipid Quantification

Highly sensitive and precise analytical methods are crucial for process development and manufacturing. In the context of LNP processing, the standard practice rarely involves the quantification of their lipid components. In this study, HPLC in combination with RP for lipid separation and CAD for lipid detection served as the basis for analytical method development. Similar methodologies for lipid quantification were already reported for individual LNP components such as PEGLs [32,33] or DSPC [29]. Further studies have presented RP-CAD methods to quantify several lipids in liposomes [34] and LNPs [19,20,35,36,37]. In general, developing such methodologies requires extensive laboratory work for improvements in chromatographic separation and precise detection.

In essence, the fundamental concept of lipid separation through RP is their relative hydrophobicity, leading to observed differences in retention times. The level of hydrophobicity is determined by a complex interplay involving lipids’ head group polarity, tail unsaturation, and the length of its alkyl chain [24]. The PEGLs DMG-PEG and DMPE-PEG have similar short-chain lengths (saturated C14) but vary in their polar head group, with DMG-PEG being more hydrophobic. Overall, higher retention times were observed for DOBAQ, DOTAP, and DSPC, with all having longer alkyl chain lengths (C18). When comparing CLs (C18 with two double bonds), DOTAP exhibited greater hydrophobicity compared to DOBAQ due to a larger hydrophobic segment and the absence of the benzoic acid group. In contrast, the tails of the amphiphilic phospholipid DSPC are fully saturated and, despite the hydrophilic phosphatidylcholine headgroup, DSPC is even more hydrophobic than both CLs. Cholesterol needs to be considered separately as it is classed as a steroid and is predominantly hydrophobic except for its polar hydroxyl head group. The observation that the retention time of cholesterol is shorter than that of DSPC is consistent with previous observations [20,34]. However, several studies have reported higher retention times for cholesterol [19,20,36,37] than for DMG-PEG, which is contrary to our findings.

This phenomenon is likely attributed to the addition of an ion-pair reagent in the mobile phase. Originally, ion-pair reagents were meant to form ion pairs with ionic compounds to improve separation and are typically used for oligonucleotides [39,40]. The ion-pair reagent enhances the analyte’s accessibility to the mobile phase as well as shields its polar groups, thereby modulating its hydrophobic interactions with the stationary phase. Li et al. [35] systematically investigated the impact of type and concentration of ion-pair reagents on the retention behavior of hydrophilic siRNAs and hydrophobic phospholipids to reduce their retention time gap. Besides retention time shifts for phospholipids, their study found peak broadening for DOTAP under certain ion-pair reagents attributable to secondary interactions with the stationary phase, which was, however, not observed in our study when using trifluoroacetic acid (TFA). Our method, utilizing a specific stationary phase and gradient design in combination with an ion-pair quantity of 0.1% TFA (*v*/*v*) and a binary gradient of acetonitril (ACN)/water, offers an alternative approach to the methods proposed by Bender et al. [20], who employed 0.15/0.1% TFA (*v*/*v*) in methanol/water, or those by Weber et al. [34], who utilized 0.2% TFA (*v*/*v*) and a ternary gradient ACN/methanol/water. ACN offers advantages over methanol due to its lower viscosity changes in gradients and its higher elution strength [41]. Apart from the ion-pair reagent, organic solvent, and gradient design, several other key chromatographic parameters critical for separation have been thoroughly explored, such as variations in the stationary phase chemistry [29,33,35] or the organic mobile phase complexity [32,36], which are, however, beyond the scope of this study.

As expected, differences in CAD-derived peak areas for identical lipid quantities were observed, which can be attributed to retention time-dependent gradient compositions. These differences, along with the non-linear behavior of the detector’s response across broad ranges [25], underscore the need for a lipid-specific calibration of the detector.

Our strategy, termed exploratory calibration, is an experimental approach and comparable to the strategies presented by Soliven et al. [28] and Tam et al. [29], broadening the apparent linear range by systematic PFV variation. While both studies [28,29] exclusively optimized the PFV for one particular substance, our approach introduces a novel aspect by targeting a single PFV applicable to all lipids, facilitating the use of the RP-CAD method for multi-component systems such as LNPs. Alternative strategies for PFV optimization may be based on empirical models [30] or mathematical transformations [31] with data acquired at PFV = 1. Our exploratory approach involving lipid standards spanning a wide range of lipid masses enables us to identify a single linearity range for all lipids. At the optimal PFV of 1.3, the signal-to-noise ratio remains acceptably low, even though increased PFVs reduce the detector’s sensitivity [28,29,30]. It should be noted that the authors decided to use a uniform lipid mass range for method calibration and validation, although a lipid quantity of up to 1200 ng for DOBAQ, DSPC, and cholesterol would have resulted in adequate linearity ( $R^{2}$ > 0.995). In this context, the lipid mass range was limited by DMPE-PEG, aligning with a reported narrower linearity range for the PEGL than for all other phospholipids [34]. A completely different approach dealing with the non-linear behavior of the detector’s response used quadratic fits for non-PEGLs across the entire calibration range instead of PFV optimization [20].

The RP-CAD method calibration and validation at PFV = 1.3 using higher amounts of data points (*p* = 9) and replicates (*n* = 3, *m* = 2) than for the exploratory calibration proved to produce linearity ( $R^{2}$ > 0.996), precision, and accuracy in the range of 10 to 400 ng for all lipids. Comparable nanogram ranges were reported [29,34], while others have developed methods in the upper nano- to lower microgram range [20,32,33]. In contrast, recent studies focusing on RP-CAD method development for LNPs have aligned the lipid mass ranges approximately with the actual lipid content in LNPs to determine the lipid-specific linearity ranges of the detector [19,36,37]. In these studies, the maximum lipid mass was two to four times the minimum lipid mass, representing a calibration range of a zero order of magnitude, whereas our calibration range of 40 times covered one order of magnitude.

Our approach with uniform linearity ranges offers the advantage of being applicable to lipid stock solutions prepared in a similar manner. However, evaluating linearity in such large calibration ranges solely based on $R^{2}$ might be misleading as percentage deviations in the lower range could be disregarded. To thoroughly validate the selected linearity range, three lipid masses, equally distributed in the selected range, were used to determine repeatability and intermediate precision—a procedure similar to that of Kim et al. [19]. As all stated deviations lie below 4%, being even lower than that reported for the other RP-CAD methods for LNPs [19,36,37], the method was considered precise. The same applies to the accuracy being comparable or even higher when compared to these methods.

In the context of robustness, a lipid mixture (DOTAP, DSPC, cholesterol, and DMG-PEG), possibly present in an LNP formulation, was used to evaluate the effect of minor method parameter variations on the detector’s response. In general, experimental procedures to determine the robustness of the RP-CAD methods have been reported to various extents [28,31,33,34]. Alternatively, Kim et al. [19] verified their method robustness by a simulation-defined method operable design region, but they underlined this theoretical robustness by additional experiments in the edge regions. As opposed to robustness studies for only one particular substance [28,33], our method has to be robust against peak overlapping. Retention time deviations observed for specific variations in separation-determining parameters, i.e., column oven temperature and flow rate, can be traced back to accompanying changes in mobile phase accessibility and hydrophobic interactions. With retention time deviations of ≤2% and unaffected baseline separation, the developed RP-CAD method is considered robust. As changes in retention times are accompanied by changed mobile phase compositions, CAD-derived peak area deviations are expected. Even though no considerable alternations in the peak areas were reported in a comparable designed robustness study with lipid mixtures [34], we observed deviations of up to 9%. To apply the here presented RP-CAD method to other HPLC systems, slightly varying method parameters, or other lipids, the generation of new experimental data for lipid-specific detector calibration and validation is strictly necessary.

### 3.2. RP-CAD Complements LNP Attributes and Reveals Process Performance

The key microfluidic process parameters, TFR and FRR [7,21,22], along with the buffer type and pH of the aqueous phase [7,21] and the lipid concentration in the organic phase [21,22], have been shown to influence the particle size and polydispersity considerably. Thus far, no holistic approach has been applied considering the effect of the process parameter variations on the lipid molar ratio, total lipid concentrations, and process performance. The presented methodology addresses lipid quantification by applying the developed RP-CAD method to the LNPs produced at different TFRs. This quantification approach complements the DLS for particle size and PDI, the ELS for surface charge, as well as a fluorescence assay for nucleic acid encapsulation.

Considering the relationship between particle size and TFR, the lowest Z-average was noted for the highest applied TFRs. The driving force of LNP formation in the presence of nucleic acids is the increase in solvent polarity, leading to a coating of the prior associated hydrophobic inverted micelles by more polar lipids [3]. In consequence, higher TFRs lead to faster polarization and hence might result in smaller particles. Other studies have also observed the inverse relationship regarding TFR and the Z-average [7,21]. While Roces et al. [7] varied the TFR within a comparable range to ours, i.e., 5 to 20 mL min^−1^, Okuda et al. [21] explored a range lower in magnitude, i.e., 0.1 to 0.5 mL min^−1^, due to the limitations imposed by their system’s pressure threshold. Conversely, Terada et al. [22] could not find a significant correlation between the Z-average and TFR for its variation in the range of 1 to 3 mL min^−1^. At a given TFR, various microfluidic systems might have slightly varying channel geometries and hence different flow characteristics compared to our system with a herringbone design for chaotic flows [42]. Therefore, the authors prioritized general trends over direct comparisons with absolute values. Moreover, other microfluidic mixing designs such as t-junction mixing or microfluidic hydrodynamic focusing are based on other mixing principles for which the TFR has to be aligned specifically, as comprehensively reviewed by Evers et al. [4].

Across LNP processing, DLS-derived data have revealed changes to higher Z-averages, with the strongest increase occurring between synthesized and dialyzed samples concomitantly with a drop in PDI. This interdependency is visualized through intensity-weighted size distributions, which are in agreement with previous studies [7]. In Terada et al. [22], similar changes in size and PDI by dialyzing in various buffers for incrementally evaluating these pH-dependent changes were also observed. During dialysis, both the removal of organic solvents and the pH change proportionally contributed to the size increase of the LNPs [9]. In first suggesting that the size increase was due to particle fusion [17], it was further proven using fluorescence-based lipid tracers [9]. Moreover, this pH-dependent fusion was thought to be restricted by the arrangement of PEGLs on the outer LNP surface, as well as the cholesterol and DSPC content [9,17]. Roces et al. [7], which further demonstrated that the size and PDI changes during dialysis are highly dependent on the lipid molar ratios and lipids selected. In our study, the short-term storage of 14 days at 2–8 °C was performed after dialysis and revealed slight, TFR-independent Z-average increases, pointing to LNP instabilities. Others conducted similar short-term stability studies in the range of hours [7] to weeks [10,21].

Although the Onpattro (patisiran) formulation has a shelf-life of three years when stored at temperatures between 2 °C and 8 °C [43], other long-term LNP stability studies for siRNA-loaded LNPs showed stable storage behavior by lyophilization or storage at −70 °C [15,44,45]. Contrary to the above-indicated instabilities of our LNPs, low-scattering, constant zeta potentials of approximately 16 mV propose colloidal stability in solution. These strong cationic zeta potentials might be attributed to the protonation of the Tris(hydroxymethyl)aminomethane (Tris) amine. Roces et al. [7] observed even higher zeta potentials for LNPs in a pH 7.4 Tris buffer, while LNPs in citrate buffer were found to be neutrally charged at pH 7.4.

To draw a more comprehensive picture of the LNP attributes across processing, the herein developed RP-CAD method was applied for lipid quantification. Our method revealed a higher DOTAP content than was theoretically aimed for, which leads to a slightly reduced N/P ratio from 5 to 4.89. However, this N/P ratio is still within the commonly used range of 3–5 for the encapsulation of comparably short nucleic acids, where typical electron-dense core structures [17] combined with high encapsulation efficiencies [10] are achieved. Further, the highest relative deviation from the expected lipid molar ratio was observed with approximately 0.6 mol% PEGL. As already mentioned, the PEGL has proven to be of great importance for the fusion behavior of LNPs [9,17]. By being located at the LNP surface, a fixed area per PEGL is given, forming the equilibrium size in dependence on the PEGL content [3,9,46]. To maintain colloidal stability during storage, it is essential to have an adequate amount of PEGL. Although Fan et al. [10] suggested a minimum of 1 mol% of a PEG_2000_ lipid to assure closely packed adjacent PEG chains to circumvent particle aggregation, stable LNPs have been observed comprising 0.5 mol% of PEGLs [9]. It has to be noted that these studies did not involve lipid quantification, and the findings are solely based on theoretical values. For our study, we have to state that, due to the low PEGL content within the LNPs, the measured peak areas are located in the lower range of our respective RP-CAD calibration curve. To definitively exclude that the PEGL content is underestimated, the approach presented by Bender et al. [20] could have been applied, calibrating a second calibration curve for the lower calibration range for PEGLs, which might have led to an even more accurate quantification.

However, across the processing, regardless of the applied TFR, all lipid contents remained stable within the LNPs, indicating lipid type- and content-independent Z-average increases. The deviations from the theoretical lipid molar ratio can hence be attributed to the lipid stock solution preparation. To the best of our knowledge, this study provides the first comprehensive assessment of the LNP’s lipid composition across processing. Considering also liposomal formulations, Weber et al. [34] investigated the lipid composition of a liposome formulation across film hydration and extrusion, and they uncovered a net lipid loss during their liposome production process. However, their PEGL quantification was affected by the peak overlapping of another phospholipid.

The RP-CAD method allows one to estimate whether robust and consistent synthesis was achieved when comparing the lipid concentrations from all synthesized LNPs. The observed scattering of lipid concentrations around target concentration indicates TFR-independent process variability during microfluidic mixing. These variations might be attributed to the procedure of syringe filling and the syringe pump setup.

In our LNP process, the RP-CAD-derived lipid loss was attributed to the purification by dialysis. Overall, we observed batch-dependent lipid recoveries that were entirely independent of the varying TFR prior to dialysis. Material absorption, possibly due to contact with plastic surfaces or membranes of the dialysis cassettes over an extended period, may constitute lipid loss. Avoiding such surfaces entirely during dialysis might be challenging, and, to the best of the authors’ knowledge, no studies have been published exploring purification performance. Moreover, the poor process performance in terms of lipid recovery could also be attributed to the lab-scale dialysis (0.3–0.5 mL) performed, as these cassettes exhibit relatively high surface-to-volume ratios and the dialyzed volumes could not be completely retrieved. Additionally, dialysis is prone to product dilution due to osmotic pressure, which was also observed to various extents in this study. To further improve process performance, larger volumes could be processed. Overall, as the deviations from the lipid molar ratio could be traced back to the lipid stock solution, and as the loss of total lipids is likely to be related to the lipid loss during dialysis, the authors considered the use of the RP-CAD method a feasible lipid quantification method.

In summary, the RP-CAD method contributes to a comprehensive analysis of LNP attributes, assists in assessing the process performance, and leads to an intensified process understanding. Furthermore, the presented method has the potential to evaluate the influence of other process- or LNP-specific parameters and to be applied to alternative processes, such as cross-flow filtration.

## 4. Materials and Methods

### 4.1. Materials and Buffers

Chemicals were purchased from Merck (Darmstadt, Germany), if not otherwise stated. All the lipids used were delivered in powder form and stored at −20 °C until usage. 1,2-Dioleoyl-3-trimethylammonium-propane (chloride salt) (DOTAP) and 1,2-Dioctadecanoyl-sn-glycero-3-phosphocholin (DSPC) were kindly provided by Lipoid (Ludwigshafen, Germany). N-(4-carboxybenzyl)-N,N-dimethyl-2,3-bis(oleoyloxy)propan-1-aminium (DOBAQ), 1,2-Dimyristoyl-rac-glycero-3-methoxypolyethylene glycol-2000 (DMG-PEG), 1,2-Dimyristoyl-sn-glycero-3-phosphoethanolamine-N-[methoxy(polyethylene glycol)-2000] (ammonium salt) (DMPE-PEG) from Avanti Polar Lipids (Alabaster, AL, USA), and cholesterol were used. Ultrapure water (Purelab ultra, ELGA LabWater, High Wycombe, UK) and HPLC grade ethanol were used for buffer and stock solution preparation. Furthermore, 25 mM acetate buffer at pH 4.0 consisted of sodium acetate trihydrate and acetic acid. Tris was used for 10 mM Tris buffer at a pH of 7.4. Buffers were pH-adjusted with 32% hydrochloric acid solution using a SenTix62 pH electrode (WTW, Weilheim, Germany) coupled to a HI 3220 pH meter (Hanna Instruments, Woonsocket, RI, USA), filtered through a 0.2 µm cellulose acetate filter (VWR International, Radnor, PA, USA). Both, LC-MS grade 0.1% TFA (*v*/*v*) in water and in ACN from Thermo Fisher Scientific Inc. (Waltham, MA, USA) and pure ACN in LC-MS grade from VWR Chemicals (VWR International) were used. NoLimits™ 20 bp DNA fragments, 5′-ATGGTGAGCAAGGGCGAGTT-3′ as forward primer, 5′-CTCGCCCTTGCTCACCATTT-3′ as reverse primer, as well as Triton™ X-100, Quant-iT™ PicoGreen™, and RiboGreen™ Assay Kits were obtained from Thermo Fisher Scientific Inc.

### 4.2. Development of the RP-HPLC-CAD Method

#### 4.2.1. Preparation of Lipid Stock Solutions

Based on preliminary experiments, 0.3 mg mL^−1^ lipid stock solutions were prepared by individually dissolving each lipid in ethanol using a Branson Ultrasonics sonifier SFX550 (Thermo Fisher Scientific Inc.). The solutions were subsequently 0.2 µm-filtered (Sartorius Stedim Biotech GmbH, Göttingen, Germany) and stored at −20 °C. Standards were prepared by dilution with ethanol, and 200 µL samples were loaded into 96-well half-area microplates (Greiner BioOne, Kremsmünster, Germany) for HPLC analysis.

#### 4.2.2. Instrumentation

An Ultimate 3000 RS HPLC system (Dionex Corporation, Sunnyvale, CA, USA) controlled by Chromeleon 6.8 (Thermo Fisher Scientific Inc.) and consisting of an HPG-3400RS pump, a WPS-3000TFC autosampler, a TCC-3000RS column compartment, a 3000RS diode array detector and a Corona Veo CAD RS. The CAD was supplied with nitrogen gas from the Corona Nitrogen 1010 (Peak Scientific Instruments GmbH, Düren, Germany) connected to the in-house compressed air system. A 2.1 × 150 mm ACQUITY^®^ BEH Phenyl column (particle size 1.7 µm, pore size 130 Å, Waters, Milford, CT, USA) was used in combination with the corresponding VanGuard™ pre-column 2.1 × 5 mm. The column temperature, autosampler temperature, flow rate, and injection volume were set to 50 °C, 8 °C, 0.3 mL min^−1^, and 8 µL, respectively. The settings for the CAD were 35 °C for the evaporation temperature and 3.6 s for the filter constant. The developed method, with 0.1% TFA (*v*/*v*) in water as mobile phase A and 0.1% TFA (*v*/*v*) in ACN as mobile phase B, started at 40%B with a 4 min hold time, followed by two linear gradients to 70%B and 100%B for 1 min and 11.25 min, respectively. After a hold time of 2 min at 100%B, a linear gradient of 1 min was used to reset to initial conditions.

#### 4.2.3. Variation of PFV and the Linearity Range

For the PFV variation study, standards were prepared in duplicates (*n* = 2) by serial dilutions of the lipid stock solutions and measured twice (*m* = 2). Measurements were recorded for each lipid with total lipid quantities of 37.5, 75, 150, 300, 600, 1200, and 2400 ng on column for PFV = 1.0, 1.1, 1.2, and 1.3, respectively. Regarding linearity, mass ranges I, II, and III were evaluated, which differed in their maximum lipid quantities (2400, 1200, and 600 ng) and thus in the amount of data points (5 ≤ *p* ≤ 7). For each range, PFV, and lipid, the linear regression was performed by the method of least squares and evaluated by the coefficient of determination $R^{2}$ . The authors referred to this procedure as exploratory calibration.

#### 4.2.4. RP-CAD Method Calibration and Validation

For the RP-CAD method calibration and validation at PFV = 1.3, all standards were prepared in triplicates (*n* = 3) and measured twice (*m* = 2). For nine-point calibration (*p* = 9), measurements were recorded for each lipid with lipid quantities of 10, 20, 40, 80, 120, 160, 200, 240, and 400 ng on column. According to the Q2(R2) guideline [38] by the ICH, the RP-CAD method was validated considering linearity, precision, accuracy, and robustness.

Regression by the method of least squares was used to evaluate the linearity with the coefficient of determination $R^{2}$ .

Precision was considered as repeatability, intermediate precision, and reproducibility. Residual standard deviations of all lipid standards with 40, 120, and 240 ng were used for repeatability. Intermediate precision and reproducibility were investigated exemplarily for DSPC. Intermediate precision was performed on two separate days for lipid standards of 40, 120, and 240 ng, while reproducibility was investigated by repeated calibration and linearity validation. Accuracy was investigated for 40, 120, and 240 ng DSPC and determined as the ratio of the measured to the expected value, expressed as the percentage recovery. Robustness was assessed by repeated injections of a lipid mixture standard with variations in the column oven temperature (50 ± 2 °C), flow rate (0.3 ± 0.01 mL min^−1^), and CAD evaporation temperature (35 ± 2 °C), and it was determined by the procentual deviation from the standard value. The lipid mixture standard contained DOTAP, DSPC, cholesterol, and DMG-PEG with a total lipid quantity of 800 ng (200 ng per lipid) on column.

### 4.3. Lipid Nanoparticle Process Characterization

#### 4.3.1. Preparation of Aqueous Nucleic Acid Stock Solution

Both reverse and forward primers were rehydrated with Tris-EDTA (TE) buffer to 0.1 mmol L^−1^, equimolar mixed, and hybridized in a C1000 Touch™ Thermal Cycler (Bio-Rad Laboratories Inc., Hercules, FL, USA). The annealing process comprised a 7 min incubation at 95 °C, step-wise cooldowns (9 °C every 5 min) to 68 °C, and a final cooldown at room temperature. Annealing and stability against 1% Triton™ X-100 were evaluated by the Quant-iT™ PicoGreen™ assay according to the manufacturer’s protocol with further adaptions. The assay was standardized with NoLimits™ 20 bp DNA fragments with three replicates of six concentrations (0, 20, 100, 300, 500, and 1000 ng mL^−1^). For both standards and samples, the assay was performed with and without 1% Triton™ X-100 treatment in 384-well black polystyrene microplates (Biozym Scientific GmbH, Hessisch Oldendorf, Germany) in a 20 µL scale. Excitation ( λ _ex_ = 480 n m ) and emission ( λ _em_ = 520 n m ) were recorded by a Spark® microplate reader (Tecan Group Ltd., Männedorf, Switzerland). The annealed primer solution was adjusted to 119 µg mL^−1^ with acetate buffer, serving as the aqueous nucleic acid stock solution with model-siRNA for all experiments.

#### 4.3.2. Microfluidics and Purification

To reach a N/P ratio of 5 after LNP synthesis, the DOTAP, DSPC, cholesterol, and DMG-PEG were dissolved (50:10:38.5:1.5 mol%) as described in Section 4.2.1 to a 12 $\;\mathrm{mg}\;{\mathrm{mL}}^{-1}$ total lipid concentration. The setup for LNP synthesis comprised a Nemesys pump (Cetoni GmbH, Korbußen, Germany) equipped with 1 mL (Innovative Labor Systeme GmbH, Stützerbach, Germany) and 10 mL glass syringes (SETsonic GmbH, Ilmenau, Germany), as well as a herringbone-structured microfluidic chip (Microfluidic ChipShop, Jena, Germany). The LNPs were synthesized in duplicates at a constant FRR of 5:1 (aqueous:organic), while the TFR was varied with 10, 15, and 20 mL min^−1^. The LNPs were dialyzed using 10 kDa Slide-A-Lyzer^®^ dialysis cassettes (Thermo Fisher Scientific Inc.) in 10 mM Tris buffer (pH 7.4) at 2–8 °C for 4 h and overnight. The final formulated LNPs were stored at 2–8 °C for 14 days.

#### 4.3.3. Lipid Concentration, Molar Ratio, and Recovery

Single lipid concentrations, a lipid molar ratio, and a total lipid recovery rate were determined by applying the developed RP-CAD method with the corresponding calibration curves. Samples were prepared in duplicates and diluted with ethanol to the final concentrations within the calibration curves. The lipid recovery rate was calculated based on the measured lipid quantities, the weights of the LNP solutions prior to and after dialysis, and the densities of the respective solution compositions regarding the ethanol-to-water content.

#### 4.3.4. Particle Size and Surface Charge

The particle size as the Z-average and the PDI of the LNPs were determined by applying DLS using a Zetasizer Nano ZSP equipped with the Zetasizer software 7.12 (both Malvern Instruments Ltd., Malvern, UK). Measurements were performed using a 633 nm laser, a 173° scatter detection angle, and a low-volume quartz batch cuvette ZEN2112 (Malvern Panalytical Ltd., Malvern, UK). The LNPs refractive index (RI) was 1.333 and the absorption was 0.01. For the dispersant, a RI of 1.341 and a viscosity of 1.919 cP were determined. Samples were diluted to a lipid concentration of 1 mg mL^−1^. Measurements were performed in duplicates with three sub-measurements, an automatic measurement duration, and a laser attenuation of 5.

To examine the surface charge as the zeta potential of the dialyzed LNP, the ELS was applied using the identical device and software but with a folded capillary cell DTS1070 (Malvern Panalytical Ltd.). For the measurements, a dielectric constant ϵ _r_ of 78.5 was set and the Helmholtz–Smoluchowski equation was applied. Each sample was measured in duplicates and five measurements per sample were performed with a minimum of 10 runs at 60 V.

#### 4.3.5. Nucleic Acid Encapsulation

The proportion of encapsulated nucleic acids in LNPs, referred to as encapsulation efficiency, was determined by the Quant-iT^TM^ RiboGreen^TM^ assay according to the manufacturer’s protocol with further adaptations. The fluorescent dye was diluted and added to the LNP sample with or without 1% Triton™ X-100. A calibration curve was obtained for each condition in the range of 20 $\;\mathrm{ng}\;{\mathrm{mL}}^{-1}$ to 1000 $\;\mathrm{ng}\;{\mathrm{mL}}^{-1}$ using NoLimits™ 20 bp DNA fragments. The samples and the calibration curves were prepared in 384-well black polystyrene microplates in duplicates and fluorescence-scanned ( λ _em_ = 520 n m , λ _ex_ = 480 n m ) using a Spark^®^ microplate reader. The encapsulation efficiency EE was calculated according to Equation (1):

(1) $$EE=\frac{m_{\mathrm{total}}-m_{\mathrm{free}}}{m_{\mathrm{total}}}*100\;\%,$$

where *m*_total_ and *m*_free_ describe the total nucleic acid mass of the sample after treatment with Triton™ X-100 and the mass of free nucleic acids outside the LNP, respectively.

### 4.4. Statistical Evaluation

Data evaluation and data visualization were performed with MATLAB^®^ R2021a (TheMathWorks Inc., Natick, MA, USA). Replicate measurements are presented as the mean ± standard deviation, and uncertainty propagation was used for the error determination. To underline the observed trends, significance was determined using Welch’s *t*-test of normally distributed data (Shapiro–Wilk test).

## 5. Conclusions

In conclusion, we present a novel holistic RP-CAD method for lipid quantification across LNP production and processing. The strategy of exploratory calibration allows for a broadening of the apparent linear range of the detector’s response by systematic optimization of the built-in PFV. With the novel aspect aiming for a single PFV applicable to all six lipids, a PFV of 1.3 was identified. Further, linearity, precision, accuracy, and robustness were proved by method calibration and validation according to the ICH Q2(R2) guideline [38]. As the RP-CAD method enables lipid quantification in pure and mixed lipid solutions, the method reveals the lipid quantities and the lipid molar ratio in a process parameter study, yielding intensified process insights. The lipid molar ratio complements other common LNP attributes—particle size, surface charge, and nucleic acid encapsulation. Furthermore, lipid recovery serves as a general indicator for process performance. The RP-CAD method uncovers a constant lipid molar ratio across production and processing, and it is entirely independent of the varied TFR during production. Overall, the developed RP-CAD method provides a foundation for integrating lipid quantification as a common analysis in LNP processes, and it bears the potential to be applied to other LNP formulations and processes such as cross-flow filtration.

## Figures and Tables

**Table 1:** Linearity. The analyzed lipids are listed with their respective slope and y-intercept of the linear regression, the corresponding coefficient of determination ( $R^{2}$ ), and the standard error of the regression ( Sy.x ). The linearity range was tested between 10 and 400 ng lipids on column.

Lipid	Retention Time	Linear Regression			
		Slope	y-Intercept	$R^{2}$	Sy.x
	min	**pA min (ng)^−1^**	pA min	-	-
DOTAP	9.69	0.0107	0.0774	0.9995	0.0010
DOBAQ	9.33	0.0153	0.1735	0.9966	0.0146
DSPC	10.13	0.0162	0.0671	0.9990	0.0050
CHOL	8.14	0.0125	0.0742	0.9988	0.0035
DMG-PEG	8.91	0.0157	−0.0973	0.9998	0.0009
DMPE-PEG	7.31	0.0094	−0.0291	0.9981	0.0031

**Table 2:** Encapsulation efficiency of the LNPs produced at different TFRs. The percentage encapsulation efficiencies ( EE ) of the LNPs produced at 10, 15, and 20 mL min^−1^ TFR.

Total Flow Rate	EE _Synthesized_	EE _Dialyzed_
mL min^−1^	%	%
10	97.07 ±1.19	98.04 ±1.42
15	98.45 ±0.47	99.88 ±0.56
20	99.99 ±0.44	100.06 ±0.49

## Data Availability

The raw data supporting the conclusions of this article will be made available by the authors without undue reservation.

## Abbreviations

The following abbreviations are used in this manuscript:

ACN	Acetonitril
CL	Cationic lipid
CAD	Charged aerosol detection
DLS	Dynamic light scattering
DMG-PEG	1,2-Dimyristoyl-rac-glycero-3-methoxypolyethylene glycol-2000
DMPE-PEG	1,2-Dimyristoyl-sn-glycero-3-phosphoethanolamine-N-[methoxy(polyethylene
	glycol)-2000] (ammonium salt)
DOBAQ	N-(4-carboxybenzyl)-N,N-dimethyl-2,3-bis(oleoyloxy) propan-1-aminium
DOTAP	1,2-Dioleoyl-3-trimethylammonium-propane (chloride salt)
DSPC	1,2-Dioctadecanoyl-sn-glycero-3-phosphocholine
ELS	Electrophoretic light scattering
FRR	Flow rate ratio
HPLC	High performance liquid chromatography
ICH	International Council for Harmonisation
LNP	Lipid nanoparticle
mRNA	Messenger RNA
PDI	Polydispersity index
PEG	Polyethylene glycol
PEGL	Polyethylene glycol lipid
PFV	Power function value
RI	Refractive index
RP	Reversed-phase
siRNA	Small-interfering RNA
TE	Tris-EDTA
TEM	Transmission electron microscopy
TFA	Trifluoroacetic acid
TFR	Total flow rate
Tris	Tris(hydroxymethyl)aminomethane
